# Changes in phenotype and differentiation potential of human mesenchymal stem cells aging in vitro

**DOI:** 10.1186/s13287-018-0876-3

**Published:** 2018-05-11

**Authors:** Yueh-Hsun Kevin Yang, Courtney R. Ogando, Carmine Wang See, Tsui-Yun Chang, Gilda A. Barabino

**Affiliations:** 1 Department of Biomedical Engineering, The City University of New York - the City College, 160 Convent Avenue, Steinman 581, New York, New York 10031 USA; 2 Department of Biomedical Engineering, The City University of New York - the City College, 160 Convent Avenue, Steinman 582, New York, New York 10031 USA; 3 Department of Biomedical Engineering, The City University of New York - the City College, 160 Convent Avenue, Steinman 142, New York, New York 10031 USA

**Keywords:** Human mesenchymal stem cell, Aging, Morphology, Proliferation, Differentiation, Adipogenesis, Osteogenesis, CD106, CD146, Gene expression

## Abstract

**Background:**

Adult mesenchymal stem cells (MSCs) hold great promise for regenerative medicine because of their self-renewal, multipotency, and trophic and immunosuppressive effects. Due to the rareness and high heterogeneity of freshly isolated MSCs, extensive in-vitro passage is required to expand their populations prior to clinical use; however, senescence usually accompanies and can potentially affect MSC characteristics and functionality. Therefore, a thorough characterization of the variations in phenotype and differentiation potential of in-vitro aging MSCs must be sought.

**Methods:**

Human bone marrow-derived MSCs were passaged in vitro and cultivated with either DMEM-based or αMEM-based expansion media. Cells were prepared for subculture every 10 days up to passage 8 and were analyzed for cell morphology, proliferative capacity, and surface marker expression at the end of each passage. The gene expression profile and adipogenic and osteogenic differentiation capability of MSCs at early (passage 4) and late (passage 8) passages were also evaluated.

**Results:**

In-vitro aging MSCs gradually lost the typical fibroblast-like spindle shape, leading to elevated morphological abnormality and inhomogeneity. While the DMEM-based expansion medium better facilitated MSC proliferation in the early passages, the cell population doubling rate reduced over time in both DMEM and αMEM groups. CD146 expression decreased with increasing passage number only when MSCs were cultured under the DMEM-based condition. Senescence also resulted in MSCs with genetic instability, which was further regulated by the medium recipe. Regardless of the expansion condition, MSCs at both passages 4 and 8 could differentiate into adipocyte-like cells whereas osteogenesis of aged MSCs was significantly compromised. For osteogenic induction, use of the αMEM-based expansion medium yielded longer osteogenesis and better quality.

**Conclusions:**

Human MSCs subjected to extensive in-vitro passage can undergo morphological, phenotypic, and genetic changes. These properties are also modulated by the medium composition employed to expand the cell populations. In addition, adipogenic potential may be better preserved over osteogenesis in aged MSCs, suggesting that MSCs at early passages must be used for osteogenic differentiation. The current study presents valuable information for future basic science research and clinical applications leading to the development of novel MSC-based therapeutic strategies for different diseases.

**Electronic supplementary material:**

The online version of this article (10.1186/s13287-018-0876-3) contains supplementary material, which is available to authorized users.

## Background

Mesenchymal stem cells (MSCs) that were initially described as colony-forming unit fibroblasts are uncommitted precursors of nonhematopoietic tissues and thus are able to give rise to several cell lineages of mesodermal origin, such as chondrocytes, osteoblasts, adipocytes, myocytes, cardiomyocytes, and tendon cells [1, 2]. Although first identified in the bone marrow, spleen, and thymus of adult mice by Friedenstein et al. [3], MSCs have been extracted from many other sources including umbilical cord blood [4, 5], synovial membrane [6], adipose tissues [6, 7], skeletal muscle [8, 9], and pancreas [10]. Among the different origins, however, bone marrow is still by far the best characterized source of MSCs due to a less invasive isolation procedure [11].

MSCs are an attractive candidate for use in regenerative medicine because of their potential for self-renewal and multipotency, as well as their trophic and immunosuppressive effects [11]. Hayflick reported that the proliferative capacity of MSCs was preserved after 27 years of cryogenic storage [12]. Clinically, MSCs have been used to improve the engraftment of hematopoietic stem cells (HSCs) post-transplantation [13] and to facilitate hematopoietic recovery from high-dose chemotherapy received by breast cancer patients when co-infused with peripheral blood progenitor cells [14]. Engraftment of allogeneic MSCs during bone marrow transplantation has led to increased bone mineral deposition and reduced frequency of bone fracture in children who suffer from severe osteogenesis imperfecta [15, 16]. Moreover, MSCs have also been researched as a vehicle for gene therapy for gut regeneration [17] and hemophilia B [18].

It has been demonstrated by several groups that the origin of MSCs can affect cellular properties and functionalities even if they are cultured under exactly the same conditions. For example, Sakaguchi et al. revealed that human MSCs derived from bone marrow exhibited the strongest osteogenic potential whereas those isolated from synovium were predominant in chondrogenesis and adipogenesis [9]. Similarly, the study carried out by Yoshimura and coworkers showed that evident osteogenesis was detected in periosteum- and muscle-derived rodent MSCs while synovium-derived cells had a stronger potential for chondrogenesis and adipogenesis [6]. This evidence also suggests that the plasticity of MSCs can be species-dependent. Furthermore, MSCs are a very rare population that only accounts for 0.01% to 0.001% of the cell numbers in bone marrow [19], and this is even lower in umbilical cord and peripheral blood [20]. Due to their unique characteristics, MSCs are usually purified by plastic adherence and remain highly heterogeneous after isolation. The low frequency and the lack of high homogeneity of freshly extracted MSC populations necessitates extensive in-vitro expansion following isolation prior to various clinical applications and essential manipulation. Thus, the use of MSCs in therapeutic treatments predominantly depends on their ability to replicate in vitro and produce progeny with strong differentiation capacity that can regenerate damaged cells, tissues, or organs. Although MSC populations may be expanded ex vivo for several generations without eliciting expression of a specific differentiated phenotype, senescence can potentially deteriorate MSC fitness to the point where the residual stem cell features are compromised and insufficient to support long-term tissue regeneration [21]. Therefore, it is important to consider the influence of in-vitro aging on cellular characteristics when designing new MSC-based therapeutic strategies.

A number of studies have been conducted to investigate human MSC aging, both in vivo and in vitro. Most of them have focused on the effects of donor age, ranging from 16 months to 90 years old, on MSC performance and have indicated that MSC frequency, population doubling rate, colony forming efficiency, and differentiation capacity decline in samples harvested from older individuals [21–24]. For in-vitro aging, since the actual age of a culture is usually presented by the number of cell population doubling (NCPD), previous research has primarily aimed to identify the upper limit of NCPD where the culture ceases to replicate and loses differentiation capability [21, 22, 25–29]. While a few groups have reported over 40 population doublings from the initial MSC passage to senescence [25, 26], others have suggested the maximum of an overall NCPD to be between 15 and 30 [21, 22, 27–29]. These studies also revealed that in-vitro aging can negatively impact the differentiation potential of MSCs, yet this was evaluated mostly on an on/off basis to determine whether or not differentiation toward a specific cell lineage can be achieved [21, 27, 28] or the percentages of differentiated cells in a population [22, 29]. The present work sought to not only distinguish the molecular and genetic phenotype of human MSCs at early and late passages within the limit of NCPD allowance (i.e., before cells cease growing or proliferating), but also characterize variations in the quality of their differentiated cells. The effects of expansion or proliferation media on MSC senescence were also investigated.

## Methods

### Cultivation of human bone marrow MSCs

MSCs extracted from the bone marrow of a 21-year-old Hispanic male donor were obtained from ATCC (Manassas, VA, USA). Cells were passaged twice before being distributed to us. To evaluate the phenotypic and morphological changes of in-vitro aging MSCs, cells were seeded on the surface of tissue culture plastics at a constant density of 1500 live cells per cm^2^ and were cultured with either of the two most commonly used MSC proliferation media inside a humidified incubator (37 °C, 5% CO_2_). The first medium consisted of low-glucose Dulbecco’s modified Eagle’s medium (DMEM), 10% fetal bovine serum (FBS), 1% penicillin/streptomycin, and 1 ng/mL basic fibroblast growth factor (FGF-2) [30–34]. The other medium was composed of minimum essential medium alpha (αMEM), 16.7% FBS, 1% penicillin/streptomycin, and 1% l-glutamine [35–37]. DMEM and αMEM experiments were conducted independently. Culture media were completely renewed at 24 h post-seeding and every 3 days thereafter. MSCs in monolayer were detached through treatment with 0.15% trypsin at 37 °C for 4 min, and collected cells were passaged at the aforementioned initial cell seeding density every 10 days. Phase-contrast images were taken during each expansion process to record cell morphology.

### MSC propagation

To determine the proliferative capability of in-vitro aging MSCs, cells collected at the end of each passage were counted using a trypan blue exclusion method. NCPD [24] and cell population doubling time (CPDT) [23] were then calculated based on the following equations:

$$ NCPD=3.33\times \log \left(\frac{N_t}{N_i}\right) $$

$$ CPDT=\left(t-{t}_i\right)\times \log \left\{2\times {\left[\log \left(\frac{N_t}{N_i}\right)\right]}^{-1}\right\} $$

where *N*_*t*_ and *N*_*i*_ are the cell numbers at a specific time point *t* (day 10) and at initial seeding (day 0), respectively.

### Flow cytometry

MSCs harvested from each passage were assessed for surface marker expression via flow cytometry. Cells were first washed with phosphate-buffered saline (PBS) and incubated with a nonspecific blocking buffer containing 1% bovine serum albumin for 30 min. After centrifugation and removal of blocking solutions, samples were treated with fluorescently conjugated mouse anti-human antibodies for 45 min. The expression of 10 surface markers was analyzed. Specifically, antibodies against Stro-1 (ab190282) and CD73 (ab106677) were purchased from Abcam (Cambridge, MA, USA); CD29 (MCA1949A647), CD34 (MCA547PE), CD44 (MCA89PE), and CD106 (MCA907F) were from Bio-Rad (Kidlington, Oxford, UK); and CD45, CD90, CD105, and CD146 (FM002) were from R&D Systems (Minneapolis, MN, USA). Amongst the surface antigens detected, CD34 and CD45 are HSC markers and thus are not expected to be expressed by MSCs while the others are MSC-specific markers [11]. Antibodies against mouse IgG were used as the negative staining isotype control. Stained cells were re-suspended in PBS and analyzed in an LSR II Flow Cytometer (BD Biosciences, San Jose, CA, USA). The size and granularity of MSCs at each passage were also evaluated using the forward and side scatter diagram in flow cytometry.

### MSC adipogenic and osteogenic differentiation

To determine the differentiation potential, MSCs at selected passages were first incubated with either DMEM-based or αMEM-based proliferation medium for 10 days as previously described and were immediately subjected to adipogenic or osteogenic conditions at the end of the expansion process without detaching the cells from the surface. The adipogenic medium was composed of high-glucose DMEM, 10% FBS, 1% penicillin/streptomycin/fungizone, 3.72 mg/mL sodium bicarbonate, 10 μL/mL insulin, 1 μM dexamethasone, 0.5 mM indomethacin, and 60 μM 3-isobutyl-1-methylxanine. In the osteogenic experiments, MSCs were fed with high-glucose DMEM supplemented with 10% FBS, 1% penicillin/streptomycin/fungizone, 3.72 mg/mL sodium bicarbonate, 50 μg/mL ascorbic acid, and 10 mM β-glycerophosphate. Cells were cultivated with either differentiation medium for up to 21 days with medium exchange every 3 days and were harvested at designated time points for assessment of gene expression and extracellular matrix (ECM) synthesis.

### Quantitative real-time polymerase chain reaction

Gene expression profiles of passaged MSCs and differentiated cells were quantified by real-time polymerase chain reaction (qPCR). Briefly, harvested cells were fixed in TRIzol, and RNA was extracted from the homogenized cell lysate through a series of rinse, elution, and centrifugation steps. The RNA samples were then reverse transcribed into cDNA using SuperScript III reagents (Life Technologies, Grand Island, NY, USA) following the manufacturer’s instructions. In the differentiation studies, the gene expression of interest was determined using Taqman qPCR probes (Life Technologies). Two adipogenic (lipoprotein lipase (LPL) and peroxisome proliferator-activated receptor γ (PPARγ)) and three osteogenic (type I collagen (Col I), runt-related transcription factor 2 (RUNX2), and alkaline phosphatase (ALP)) markers were examined. cDNA derived from MSCs at passages 4 (P4) and 8 (P8) was analyzed by a customized qPCR array (Qiagen, Hilden, Germany) that implements the SYBR Green real-time telomeric repeat amplification protocol. Eight stemness genes and fifty potential MSC markers were screened (Table 1). The fluorescent signals were amplified and detected using a StepOnePlus sequence detector (Life Technologies). The reaction consisted of an initial enzyme activation for 10 min at 95 °C, followed by 40 cycles of 15 s at 95 °C and 1 min at 60 °C. The cycle threshold (Ct) value for each sample was averaged from triplicates. A 2^–ΔΔCt^ approach [38] was used where the fluorescent signals were normalized to the corresponding housekeeping gene (glyceraldehyde-3-phosphate dehydrogenase (GAPDH)). For easy comparison, a heatmap was created for each expansion condition (i.e., DMEM-based and αMEM-based groups) based on the qPCR array data.

**Table 1:** Stem cell genes analyzed in real-time polymerase chain reaction array

	Gene name
Stemness genes	FGF2, INS, LIF, POU5F1, SOX2, TERT, WNT3A, ZFP42
Mesenchymal stem cell genes	ALCAM, ANPEP, ANXA5, BDNF, BGLAP, BMP2, BMP7, CASP3, CD44, COL1A1, CSF2, CSF3, CTNNB1, EGF, ENG, ERBB2, FUT1, FUT4, FZD9, GTF3A, HGF, ICAM1, IFNG, IGF1, IL10, IL1B, IL6, ITGA6, ITGAV, ITGB1, KDR, KITLG, MCAM, MMP2, NES, NGFR, NT5E, NUDT6, PDGFRB, PIGS, PROM1, PTPRC, SLC17A5, TGFB3, THY1, TNF, VCAM1, VEGFA, VIM, VWF

### Histology

Samples collected in the differentiation experiments were fixed in 10% formalin for 1 h at room temperature and were processed for histological evaluation. Adipogenic samples were first treated with 60% isopropanol for 5 min, followed by 5-min incubation with 1.8% w/v Oil Red O dissolved in isopropanol to stain lipids. Osteogenic specimens were incubated with 2% w/v Alizarin Red S dissolved in deionized water for 45 min to visualize calcium. Several rinses with water were made between each step. Color images were captured under a light microscope (Nikon Eclipse Ti, Tokyo, Japan).

### Statistical analyses

Statistical data are presented as means ± one standard deviation. Statistical analyses were performed by Student’s *t* test for comparison between two groups or by one-way analysis of variance in conjunction with a Bonferroni post-test for multiple comparisons with significance set at a *p* value of less than 0.05.

## Results

### Reduced proliferative capability of in-vitro aging MSCs

**Fig. 1:**
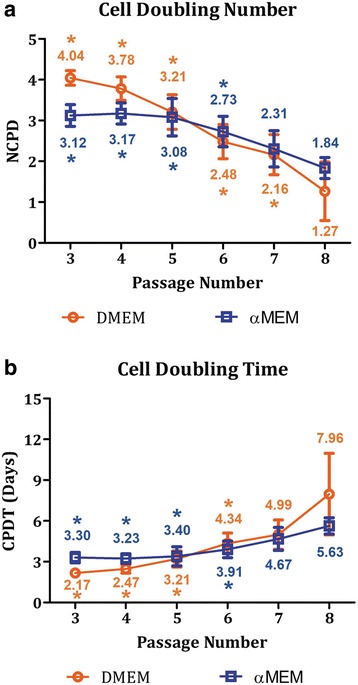
Propagation of in-vitro aging MSCs. **a** Numbers of cell population doubling (NCPD) decreased by 69% and 41% from P3 to P8 in the DMEM and αMEM groups, respectively. **b** As a result of the reduced proliferative capability, MSC population doubling time (CPDT) increased over time in both groups. Numbers represent the mean values. *n* = 5–8; *p < 0.05, versus the corresponding P8-MSCs cultured under the same expansion condition. αMEM, minimum essential medium alpha; DMEM, Dulbecco’s modified Eagle’s medium

MSCs were processed for subculture every 10 days, and NCPD and CPDT were measured at the end of each passage (Fig. 1). Although MSCs nourished with the DMEM-based expansion medium exhibited stronger proliferative capability in the initial passages compared to the other group, the average NCPD started to fall behind after P6 (Fig. 1a). Specifically, NCPD in the DMEM cultures dropped significantly from 4.04 in P3 to 1.27 in P8. While MSCs exposed to the αMEM-based culture condition were able to maintain a similar cell doubling rate up to P5 with an average NCPD of ~ 3.1, the value gradually declined thereafter and reached 1.84 in P8. Overall, NCPD decreased by 69% and 41% from P3 to P8 and accumulative NCPD was around 16.9 and 16.2 at the end of P8 in the DMEM and αMEM groups, respectively. Conversely, CPDT extended from 2.17 to 7.96 days in the DMEM group and from 3.3 to 5.63 days in the αMEM group as the passage number increased from 3 to 8 (Fig. 1b).

### Increased morphological inhomogeneity of in-vitro aging MSCs

**Fig. 2:**
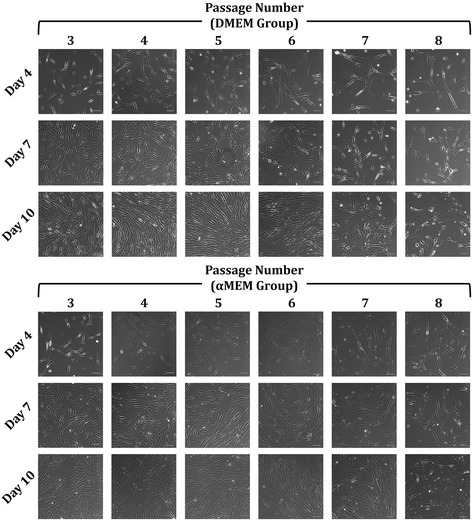
Morphological representation of in-vitro aging MSCs. Phase-contrast images of MSCs at P3 through P8 exposed to the DMEM-based (top panel) and αMEM-based (bottom panel) culture conditions were captured on days 4, 7, and 10. Scale bars = 100 μm. αMEM, minimum essential medium alpha; DMEM, Dulbecco’s modified Eagle’s medium

**Fig. 3:**
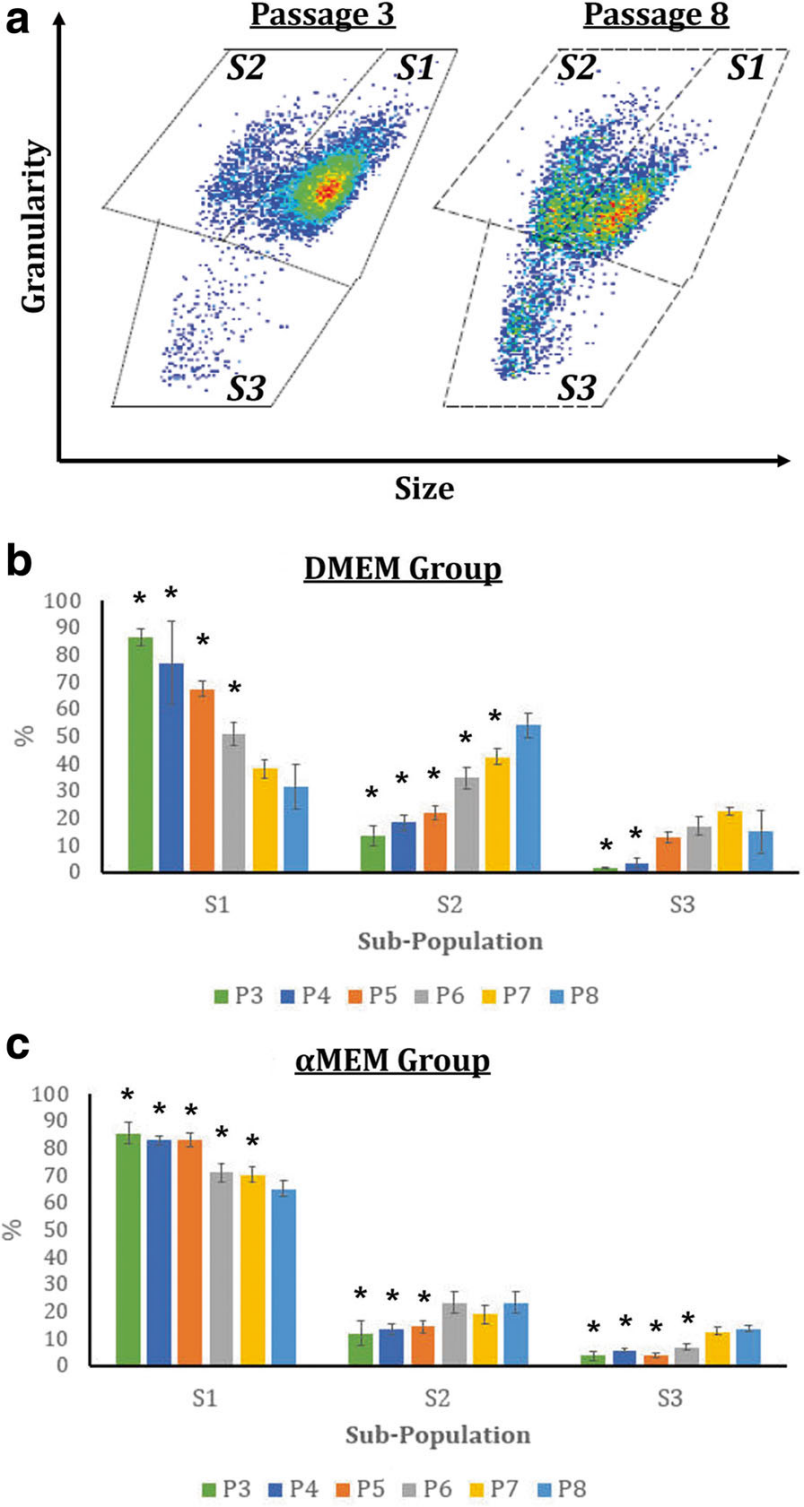
Morphological evaluation of in-vitro aging MSCs. **a** Passaged MSCs were divided into three subpopulations, *S1*, *S2*, and *S3*, based on the forward (size) and side (granularity) scatter parameters in flow cytometry analysis. **b**, **c** Cell counts in the main subpopulation, *S1*, decreased by 64% and 24% from P3 to P8 in the DMEM (**b**) and αMEM (**c**) groups, respectively. *n* = 20; *p < 0.05, versus the corresponding P8-MSCs within the same subpopulation. αMEM, minimum essential medium alpha; DMEM, Dulbecco’s modified Eagle’s medium

Microscopic images demonstrate that while most of the MSCs cultured with either medium formulation maintained their normal spindle shape in the early passages up to P5, those at later passages exhibited less concordant cell morphologies with some of them possessing irregular flattened geometry and enlarged size (Fig. 2). Morphological distribution of in-vitro aging MSCs was determined by the size-granularity diagram in flow cytometry where cells were divided into three subpopulations (*S1*, *S2*, and *S3*) as shown in Fig. 3a. In the early passages, MSC populations were more concentrated and primarily occupied the subpopulation *S1* region. As the passage number increased, the main MSC population started shifting from *S1* to *S2* and *S3* in both groups. Specifically, in the DMEM group (Fig. 3b), *S1* downsized by 64% from P3 (86.7%) to P8 (31.4%) whereas *S2* and *S3* populations rose from 13.4% and 1.6% in P3 to 54.1% and 15.0% in P8, respectively. Similarly, the size of *S1* in the αMEM group decreased over time from 85.5% (P3) to 65.1% (P8) while both *S2* and *S3* expanded as MSCs aged in vitro (Fig. 3c). Morphologically, more inhomogeneous MSC populations were detected in the cultures with a passage number of 6 or higher in both DMEM and αMEM groups.

### Variations in CD106 and CD146 levels

**Fig. 4:**
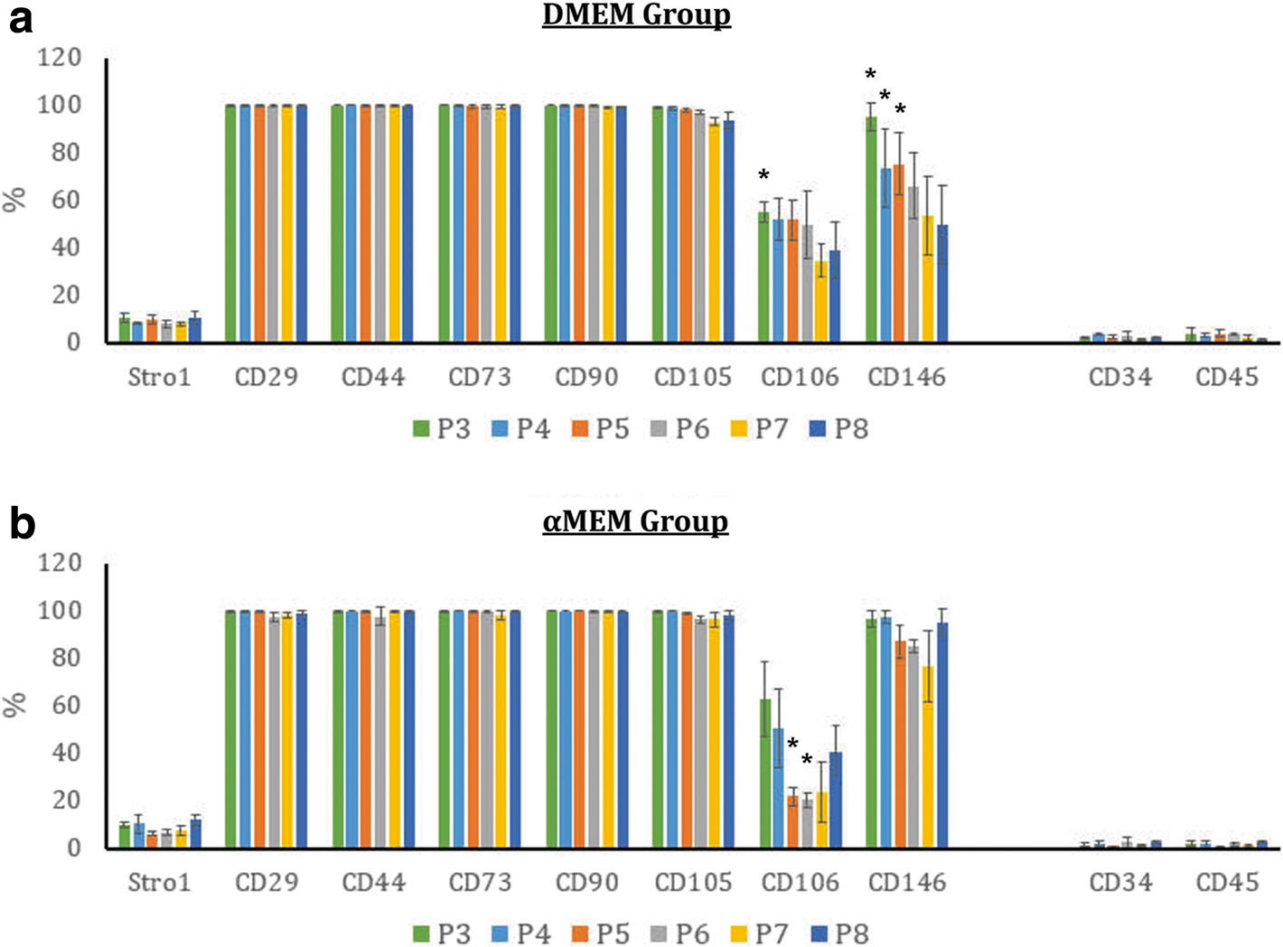
Surface marker expression of in-vitro aging MSCs. MSCs were cultivated with either DMEM-based (**a**) or αMEM-based (**b**) expansion media and were analyzed by flow cytometry for the expression of specific MSC positive (Stro-1, CD29, CD44, CD73, CD90, CD105, CD106, CD 146) and negative (CD34, CD45) surface antigens. *n* = 4–6; *p < 0.05, versus the corresponding P8-MSCs cultured under the same expansion condition within the same surface marker analysis. αMEM, minimum essential medium alpha; DMEM, Dulbecco’s modified Eagle’s medium

Surface antigen expression of passaged MSCs was assessed by flow cytometry (Fig. 4). Eight MSC-specific markers, Stro-1, CD29, CD44, CD73, CD90, CD105, CD106, and CD146, and two HSC markers, CD34 and CD45, were evaluated. While most of the MSC-positive markers including CD29, CD44, CD73, CD90, and CD105 were highly expressed by at least 96.6% of the cells across different passages in both DMEM (Fig. 4a) and αMEM (Fig. 4b) groups, expression of CD106, CD146, and Stro-1 was somehow variable. Specifically, CD106 levels oscillated throughout the entire expansion process with the average values ranging from 34.6% (P7) to 55.4% (P3) in the DMEM group and from 20.2% (P6) to 62.7% (P3) in the αMEM group. The size of CD146^+^ populations stayed similar at all point in the αMEM group (76.5–97.3%) but continued to shrink in the DMEM group as the passage number increased (95.1% in P3 versus 49.7% in P8). Despite being an MSC-positive marker, Stro-1 levels were extremely low compared with the other positive markers in any passages in both groups, and only about 9% of passaged MSCs contained Stro-1. Moreover, the majority of MSCs passaged under either condition remained negative for both CD34 and CD45.

### Gene expression profiles of in-vitro aging MSCs affected by medium composition

**Fig. 5:**
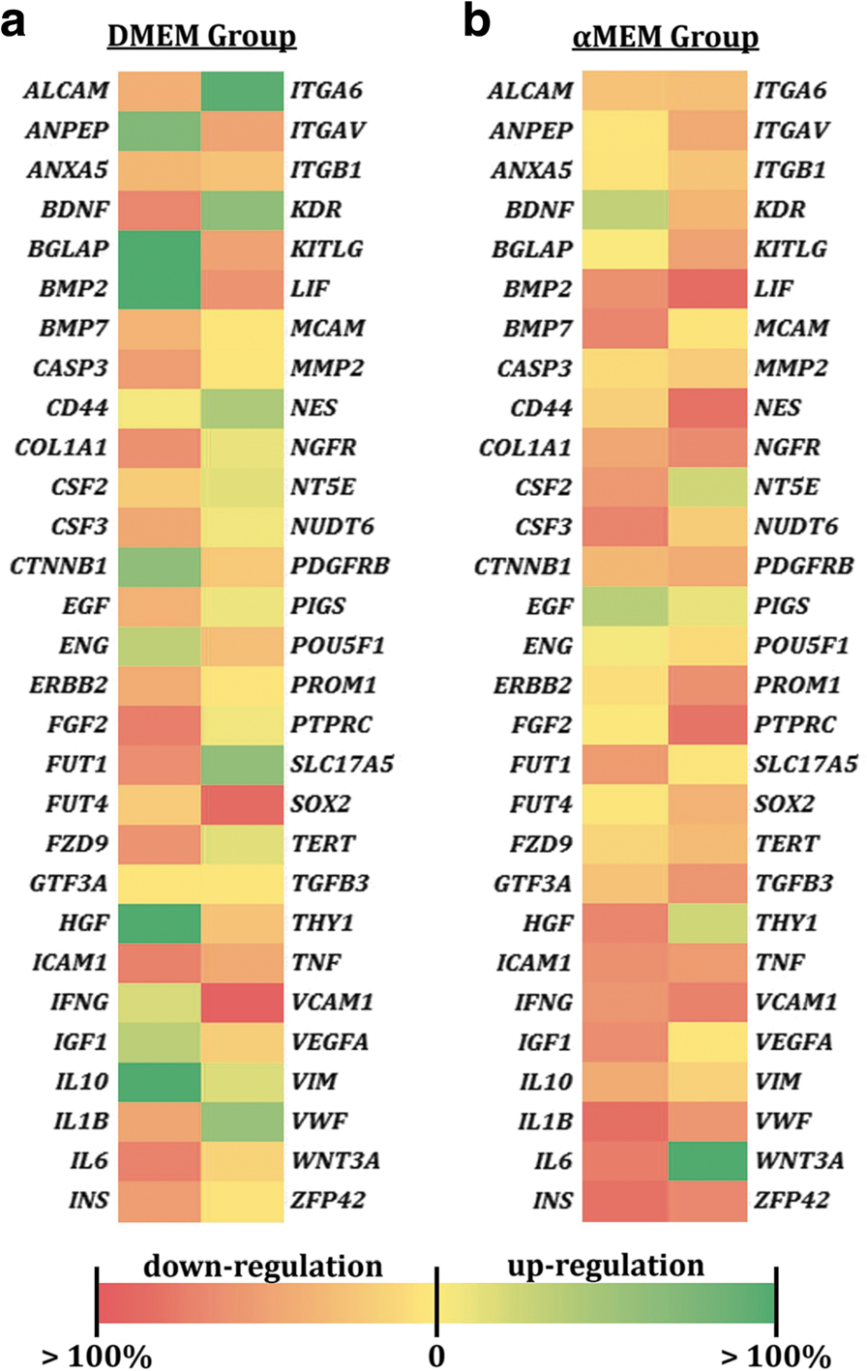
Gene expression profiles of P8-MSCs in comparison with P4-MSCs. MSCs were cultivated with either DMEM-based (**a**) or αMEM-based (**b**) expansion media and were analyzed by qPCR array for the expression of eight stemness (FGF2, INS, LIF, POU5F1, SOX2, TERT, WNT3A, ZFP42) and fifty MSC-specific genes. GAPDH was used as the reference gene, and the corresponding P4-MSCs were used as the control groups. αMEM, minimum essential medium alpha; DMEM, Dulbecco’s modified Eagle’s medium

A qPCR array was used to determine gene expression profiles of passaged MSCs (Fig. 5). The outcome is quite distinct when cells were cultured with different proliferation media. In the DMEM group, four stemness (FGF2, INS, LIF, and SOX2) and eight MSC-specific (BDNF, CASP3, COL1A1, FUT1, FZD9, ICAM1, IL6, and VCAM1) genes decreased by more than 50% in P8 versus P4, and another 11 markers reduced by 30–50% (Fig. 5a). When MSCs were exposed to the αMEM-based culture condition, three stemness (INS, LIF, and ZFP42) and 20 MSC-specific (BMP2, BMP7, CSF2, CSF3, FUT1, HGF, ICAM1, IFNG, IGF1, IL1B, IL6, KITLG, NES, NGFR, PROM1, PTPRC, TGFB3, TNF, VWF, and VCAM1) genes were downregulated by more than 50% in P8 versus P4, and another eight markers lowered by 30–50% (Fig. 5b). Interestingly, amongst the genes examined, only one of them (WNT3A) in the αMEM group, but 10 (ANPEP, BGLAP, BMP2, CTNNB1, HGF, IL10, ITGA6, KDR, SLC17A5, and VWF) in the DMEM group were upregulated by more than 50% in P8-MSCs in comparison with P4-MSCs.

### Better preservation of adipogenic over osteogenic potential by in-vitro aging MSCs

**Fig. 6:**
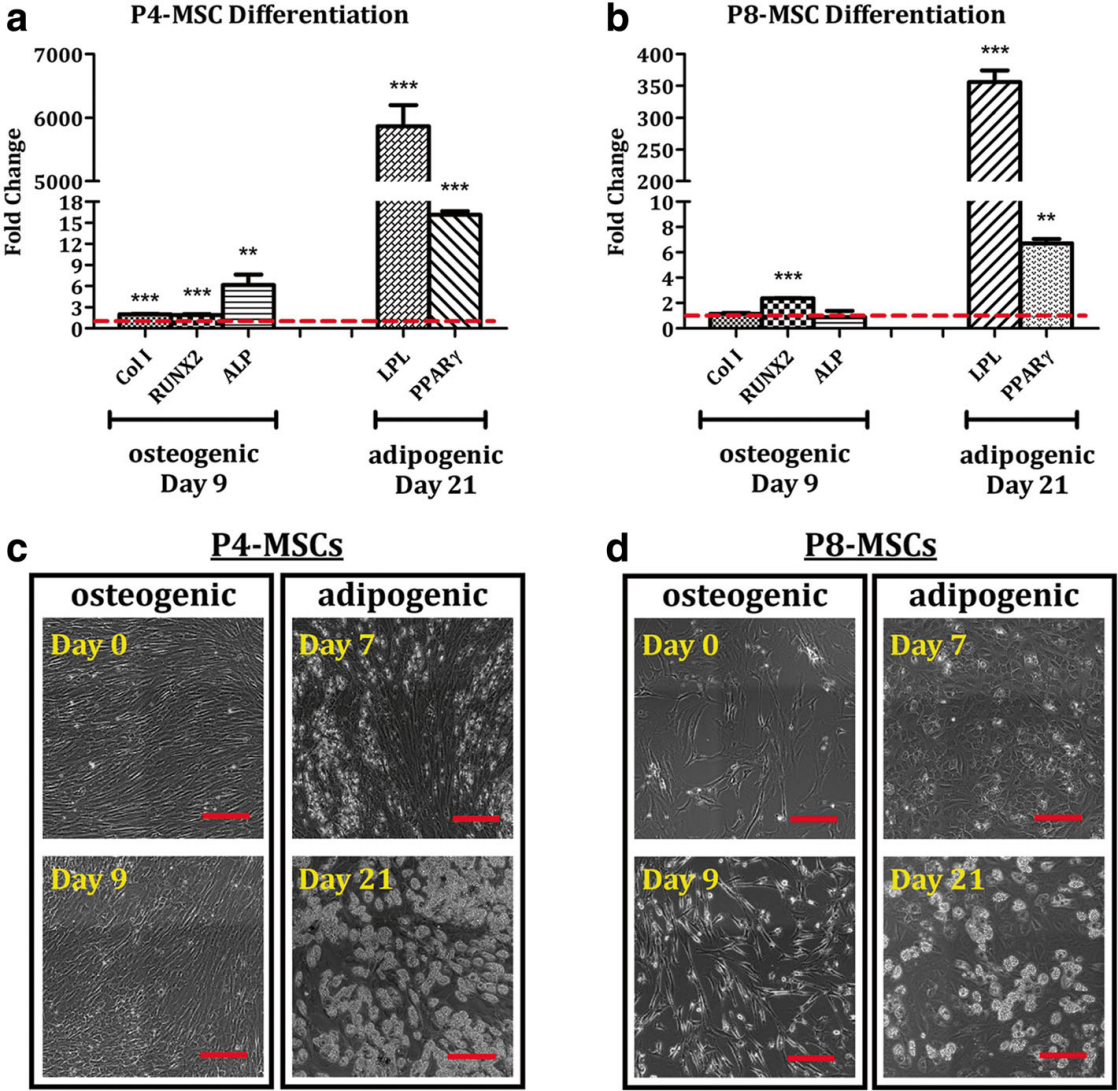
Adipogenic and osteogenic differentiation of MSCs derived from the DMEM-based expansion condition. MSCs at P4 (**a**, **c**) or P8 (**b**, **d**) were cultivated with either osteogenic differentiation media for 9 days or adipogenic differentiation media for 21 days. **a**, **b** Expression of osteogenic (Col I, RUNX2, ALP) and adipogenic (LPL, PPARγ) genes was determined by qPCR with GAPDH as the reference gene and nonconditioned MSCs at the corresponding passages as the control groups (red dashed lines). *n* = 4; **p < 0.01, ***p < 0.001, versus nonconditioned P4-MSCs or P8-MSCs. **c**, **d** Phase-contrast images of osteogenic and adipogenic samples were captured at the designated time points. White clusters in the adipogenic images represent lipids produced by the cells. Scale bars = 250 μm. ALP, alkaline phosphatase; Col I, type I collagen; LPL lipoprotein lipase; MSC, mesenchymal stem cell; P, passage; PPARγ, peroxisome proliferator-activated receptor γ; RUNX2, runt-related transcription factor 2

Adipogenic and osteogenic differentiation capacities of passaged MSCs were investigated and the results derived from P4 (early passage) and P8 (late passage) cells were reported. When the populations were expanded under the DMEM-based condition, both P4-MSCs and P8-MSCs could differentiate into adipocytes after 21 days of induction as evidenced by elevated expression of LPL and PPARγ genes and synthesis of lipid vacuoles (Fig. 6). Specifically, in comparison with nonconditioned MSCs at the corresponding passages, levels of LPL and PPARγ increased by 5865-fold and 16.1-fold, respectively, in the differentiated P4 cells (Fig. 6a) and by 356-fold and 6.7-fold, respectively, in the differentiated P8 cells (Fig. 6b). The cells undergoing adipogenesis not only changed their morphologies to a polygonal or round shape, but also produced abundant lipids (Fig. 6c, d). On the contrary, P4-MSCs treated with osteogenic differentiation media for 9 days exhibited significantly stronger expression of Col I (1.98-fold), RUNX2 (1.88-fold), and ALP (6.14-fold) genes than those in the undifferentiated state (Fig. 6a) while only the RUNX2 signal (2.37-fold) was amplified in the P8 group (Fig. 6b). Moreover, cells with a similar appearance uniformly distributed in the P4 osteogenic cultures (Fig. 6c) as opposed to those at P8 which had relatively discordant cell shapes.

**Fig. 7:**
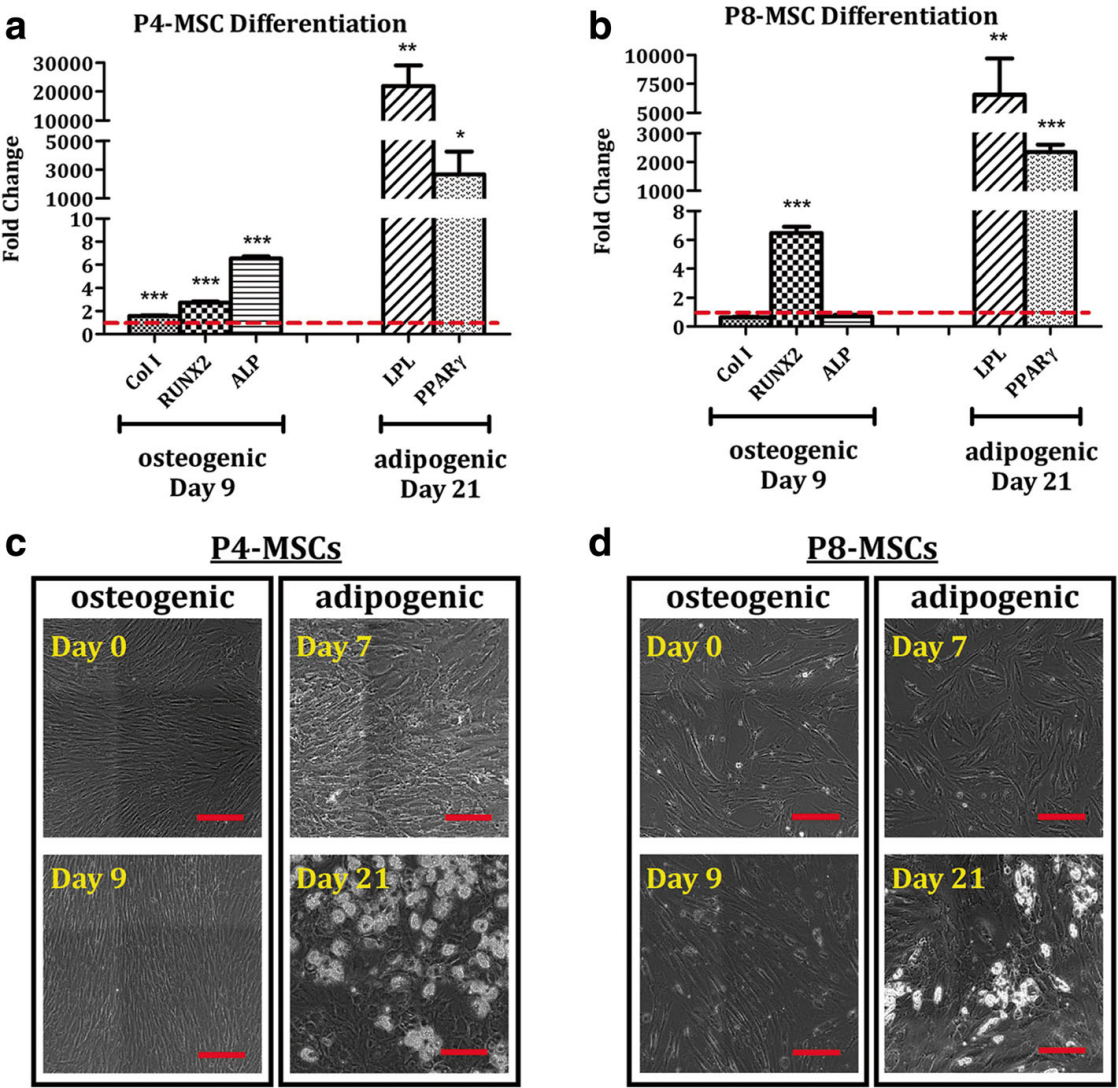
Adipogenic and osteogenic differentiation of MSCs derived from the αMEM-based expansion condition. MSCs at P4 (**a**, **c**) or P8 (**b**, **d**) were cultivated with either osteogenic differentiation media for 9 days or adipogenic differentiation media for 21 days. **a**, **b** Expression of osteogenic (Col I, RUNX2, ALP) and adipogenic (LPL, PPARγ) genes was determined by qPCR with GAPDH as the reference gene and nonconditioned MSCs at the corresponding passages as the control groups (red dashed lines). *n* = 4; *p < 0.05, **p < 0.01, ***p < 0.001, versus non-conditioned P4-MSCs or P8-MSCs. **c**, **d** Phase-contrast images of osteogenic and adipogenic samples were captured at the designated time points. White clusters in the adipogenic images represent lipids produced by the cells. Scale bars = 250 μm. ALP, alkaline phosphatase; Col I, type I collagen; LPL lipoprotein lipase; MSC, mesenchymal stem cell; P, passage; PPARγ, peroxisome proliferator-activated receptor γ; RUNX2, runt-related transcription factor 2

A similar outcome was demonstrated in the αMEM group (Fig. 7). In gene expression, LPL and PPARγ signals were amplified in both P4 (21,868-fold in LPL, 2666-fold in PPARγ; Fig. 7a) and P8 (6550-fold in LPL, 2345 fold in PPARγ; Fig. 7b) adipogenic cultures. Levels of Col I, RUNX2, and ALP were, respectively, 1.58-fold, 2.73-fold, and 6.53-fold higher in osteogenically differentiated cells than nonconditioned P4-MSCs (Fig. 7a) whereas incubation with osteogenic differentiation media only stimulated RUNX2 expression (6.47-fold) in the P8 samples (Fig. 7b). Phase-contrast images demonstrate morphological changes of P4-MSCs (Fig. 7c) and P8-MSCs (Fig. 7d) during adipogenic and osteogenic induction.

### Compromised properties of cells differentiated from aged MSCs

**Fig. 8:**
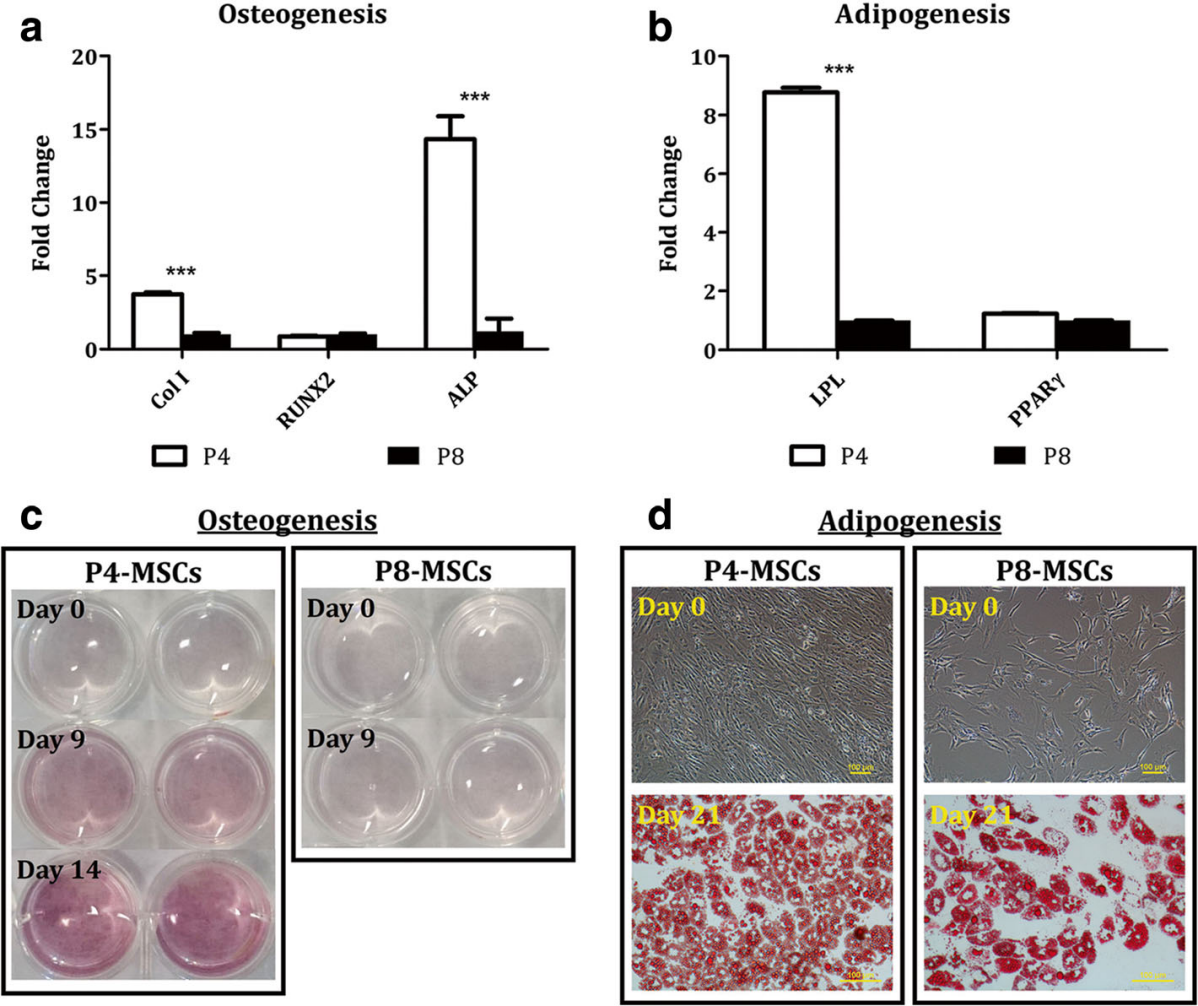
Differentiation capacity of P4-MSCs versus P8-MSCs derived from the DMEM-based expansion condition. MSCs at P4 or P8 were cultivated with either osteogenic differentiation media for up to 14 days (**a**, **c**) or adipogenic differentiation media for 21 days (**b**, **d**). **a** Gene expression of osteogenic samples was assessed on day 9. **b** Gene expression of adipogenic samples was assessed on day 21. In qPCR, GAPDH was used as the reference gene, and the corresponding P8 samples were used as the control groups. *n* = 4; ***p < 0.001, versus the corresponding P8 groups. **c** Osteogenic cultures were stained histologically with Alizarin Red S and calcium is shown in a red color. **d** Adipogenic cultures were stained histologically with Oil Red O and lipids are shown in a red color. Scale bars = 100 μm. ALP, alkaline phosphatase; Col I, type I collagen; LPL lipoprotein lipase; MSC, mesenchymal stem cell; P, passage; PPARγ, peroxisome proliferator-activated receptor γ; RUNX2, runt-related transcription factor 2

Gene expression and ECM synthesis of cells differentiated from P4-MSCs and P8-MSCs were directly compared. In the DMEM group, levels of Col I and ALP in the P4 cells were respectively 3.72-fold and 14.3-fold higher than those in the P8 cells after 9-day osteogenic induction while RUNX2 expression was comparable (Fig. 8a). Additionally, calcium deposition was barely detectable in the P8 samples whereas the intensity of calcium staining increased over time in the P4 cultures (Fig. 8c). During adipogenesis, P4-MSCs differentiated into cells that had 8.77-fold stronger LPL gene expression (Fig. 8b) and synthesized more lipid vacuoles (Fig. 8d) after 21 days in culture as opposed to the P8 group.

**Fig. 9:**
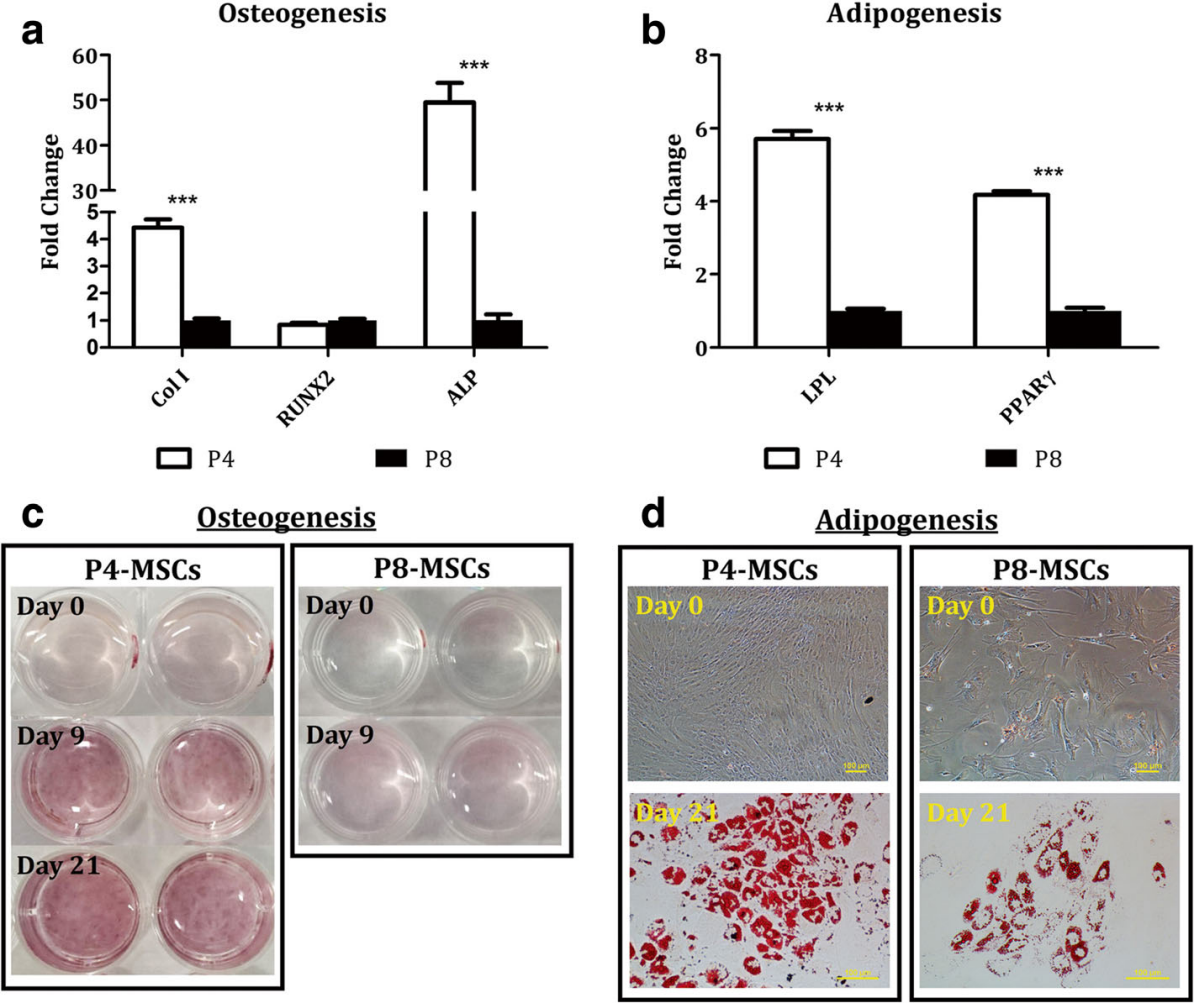
Differentiation capacity of P4-MSCs versus P8-MSCs derived from the αMEM-based expansion condition. MSCs at P4 or P8 were cultivated with either osteogenic (**a**, **c**) or adipogenic (**b**, **d**) differentiation media for up to 21 days. **a** Gene expression of osteogenic samples was assessed on day 9. **b** Gene expression of adipogenic samples was assessed on day 21. In qPCR, GAPDH was used as the reference gene, and the corresponding P8 samples were used as the control groups. *n* = 4; ***p < 0.001, versus the corresponding P8 groups. **c** Osteogenic cultures were stained histologically with Alizarin Red S and calcium is shown in a red color. **d** Adipogenic cultures were stained histologically with Oil Red O and lipids are shown in a red color. Scale bars = 100 μm. ALP, alkaline phosphatase; Col I, type I collagen; LPL lipoprotein lipase; MSC, mesenchymal stem cell; P, passage; PPARγ, peroxisome proliferator-activated receptor γ; RUNX2, runt-related transcription factor 2

Osteogenic differentiation of P4-MSCs that were initially expanded in the αMEM-based medium led to elevated expression of Col I (4.43-fold) and ALP (52.8-fold) genes and an equal RUNX2 level compared with P8-MSCs (Fig. 9a). Similarly, LPL and PPARγ signals of the P4 adipogenic samples were respectively 5.71-fold and 4.18-fold stronger than the P8 specimens (Fig. 9b). Although some calcium staining was detected on day 9 in the P8 osteogenic group, osteoblast-like cells derived from P4-MSCs deposited relatively more calcium (Fig. 9c). When exposed to adipogenic conditions, both P4-MSCs and P8-MSCs were able to produce lipids, though more lipid vacuoles were observed in the P4 group (Fig. 9d). Histological evaluation also demonstrated that most of synthesized lipids accumulated in the cytoplasm (Figs. 8d and 9d).

## Discussion

Cell aging is a complex process and current thoughts on mechanisms underlying senescence have mainly focused on the loss of telomere length. In a preliminary study, the relative telomere length of MSCs at different passage numbers was measured using the Cawthon’s qPCR method [39] and a decreasing trend was observed over time in both DMEM (Additional file 1: Figure S1C) and αMEM (Additional file 1: Figure S1D) groups. Although different telomere lengths of MSCs have been reported in the literature, ranging from 7 kb to 13 kb depending on donor age, all these studies concluded telomere loss of up to 2 kb when MSCs are passaged in vitro [21, 29, 40]. Furthermore, some groups have also demonstrated that MSCs harvested from telomerase knock-out animals have compromised replicative capacity [41] and can completely lose the ability to differentiate, even in early passages [42]. On the contrary, MSCs transduced with telomerase reverse transcriptase exhibited prolonged proliferative lifespan and enhanced osteogenic potential [43, 44]. However, the efficiency of this genetic engineering approach to the generation of large MSC populations for clinical purposes is still unclear. Another possible contributing factor to cell senescence is the p16^INK4A^ gene, the expression of which has been shown to be closely associated with MSC aging and to be silenced by DNA methylation during in-vitro expansion, leading to chromosomal aberrations [45]. Recently, Blázquez-Prunera et al. formulated a culture medium with a xeno-free supplement derived from human plasma that held promise for the maintenance of MSC phenotype, multipotency, and genetic stability during in-vitro passage [46]; however, more studies on this defined xeno-free human plasma fraction are necessary to confirm its effectiveness.

In the present study, we designed a series of experiments to explore changes in cell morphology, proliferative rate, surface marker and gene expression, and differentiation capability of human MSCs undergoing in-vitro aging and included two recipes for MSC proliferation media that are the most commonly used in the field. Given that the initial MSC batch we received were P2 cells and could achieve an additional 21.15 doublings as recommended by the vendor (documents not shown; ATCC lot no. 62535836), we evaluated MSCs with an accumulative NCPD of up to 16.9 in the DMEM group and 16.2 in the αMEM group. We estimated that the overall NCPD of the cells examined in this study should stay under 25 which falls in the effective range (15–30) reported by several other groups [21, 22, 27–29], assuming the individual NCPD in the first two passages, i.e., P1 and P2, did not deviate too much from our P3 data shown in Fig. 1a. Our results indicate that the DMEM-based expansion condition better facilitated MSC proliferation in the early passages (P3 and P4), but NCPD in this group rapidly dropped thereafter. A relatively steady NCPD curve was observed in the αMEM-supplemented culture. Overall, passaged MSCs exhibited a tendency to lose the ability of mitotic divisions during in-vitro aging as evidenced by the decrease in NCPD by 69% and 41% from P3 to P8 in the DMEM and αMEM groups, respectively (Fig. 1a), which is in agreement with previous investigations [21, 22, 25, 27–29]. As a result of compromised replicative capacity, CPDT rose over time in both groups when the passage number increased (Fig. 1b). Morphologically, instead of a uniform fibroblast-like spindle shape observed in the early cultures, some of MSC populations displayed enlarged and flattened appearance in the later passages (Fig. 2). It is believed that an increase in cell size is highly associated with senescence in vitro [12, 47]. Our flow cytometry analysis further confirms that in-vitro aging can contribute to a more scattered distribution of MSC populations in the late passages, which substantiates morphological abnormality and inhomogeneity (Fig. 3). We also found that the αMEM-based proliferation medium may preserve MSC morphology for a relatively longer period as opposed to the DMEM group (Fig. 3b and c).

The expression of eight MSC and two HSC surface markers was assessed (Fig. 4). As expected, passaged MSCs remained negative for both CD34 and CD45 throughout the entire expansion process. At least 96.6% of the MSC populations expressed CD29, CD44, CD73, CD90, and CD105 antigens regardless of culture condition and passage number. Stro-1 levels were found to be lower than 10% in any passages when MSCs were cultivated with either DMEM-based or αMEM-based proliferation medium. It is thought that Stro-1 is unlikely to be a common MSC marker and may only be used for initial MSC isolation because its expression is not exclusive to MSCs and can quickly disappear during in-vitro expansion [11, 48]. In addition, passaged MSCs displayed variable CD106 levels in both groups that were not proportionate to passage number. It has been previously revealed that CD106^+^ and CD106^−^ cells coexist in MSC populations and both populations possess multipotent capacity, although CD106^+^ MSCs have more immunoregulatory effects on T helper subsets [49] and stronger potential for capillary tubular formation than CD106^−^ cells [50]. Therefore, oscillated CD106 expression may not be attributed to in-vitro MSC aging. A decreasing trend in the CD146 signal was detected only in the DMEM-supplemented samples (Fig. 4a). As demonstrated by Sacchetti et al., osteoprogenitor cells that regenerate bone and organize hematopoietic microenvironments are highly positive for CD146 [51], and thus the reduction in CD146 may subsequently impair the osteogenic potential of aged MSCs in the DMEM group when compared with the αMEM group. We further analyzed the surface marker expression in each of the three MSC subpopulations, *S1*, *S2*, and *S3*, as defined in Fig. 3a; however, there was no obvious correlation between the two parameters (data not shown), implying that surface marker expression may not be affected by morphological changes of in-vitro aging MSCs.

Expression of over 30 stem cell genes evaluated in this study differed in MSCs derived from early (P4) and late (P8) passages (Fig. 5). While qPCR signals from about one-third of the altered genes in the DMEM group increased in P8 versus P4, only one gene was upregulated in the αMEM group. This outcome suggests that: 1) in-vitro senescence can lead to genetic instability of MSCs; and 2) gene expression of aging MSCs is highly regulated by culture media that are selected to expand the populations and thereby should be considered carefully. It is noteworthy that the genetic instability of aging MSCs may subsequently result in altered levels of ECM components and growth factors secreted by the cells; this requires further investigation.

Our results also show that while MSCs at early passages (P4) derived from either expansion condition were able to develop into adipocyte-like and osteoblast-like cells upon proper stimulation, lineage-dependent differentiation capacity was detected in cells at later passages (P8) (Figs. 6, 7, 8 and 9). Adipogenic differentiation was achieved in both P4 and P8 cultures as evidenced by elevated expression of adipocyte-associated genes (LPL and PPARγ) and production of abundant lipid vacuoles in comparison with unstimulated MSCs (Figs. 6 and 7). Although expression of LPL and/or PPARγ genes was somehow compromised in the P8 samples compared to the corresponding P4 groups, it did not affect the ability of cells differentiated from P8-MSCs to synthesize lipids (Figs. 8 and 9). During osteogenesis, however, cells derived from P8-MSCs had relatively discordant morphologies and only RUNX2 levels were higher than the controls after 9 days in culture (Figs. 6 and 7). Calcium deposition as well as qPCR signals from both Col I and ALP genes reduced in P8 versus P4, while RUNX2 expression was comparable (Figs. 8 and 9). Since RUNX2 is a key transcription factor involved in the early phase of osteogenesis [52, 53], it may require more time for the P8 samples to complete the differentiation process. However, spontaneous cell detachment was observed during osteogenesis in the P8 cultures in both DMEM and αMEM groups such that the differentiation induction only lasted up to 10 days. Even in the early passages, cell detachment also occurred in the DMEM group and the P4 osteogenic experiments could only run for 2 weeks. The reasons behind this spontaneous cell detachment remain unknown and further investigations are necessary. Histologically, although the intensity of calcium staining was extremely weak in the P8 osteogenic cultures, some accumulation was detected in the αMEM group (Fig. 9c) whereas no stains were viewed in the DMEM group (Fig. 8c). In summary, despite some contradictory results that indicated quick dissipation of adipogenic capacity of aged MSCs [27–29], our findings suggest that adipogenic potential may be better preserved over osteogenesis in human MSCs undergoing in-vitro aging. This outcome is consistent with the in-vivo observations that showed an increase in adipose tissue volume, but decreased bone formation, when MSCs senesce [54].

## Conclusions

The current work presents a thorough investigation on variations in phenotype and differentiation capability of in-vitro aging human MSCs derived from bone marrow. We found that MSCs subjected to extensive in-vitro passage can experience morphological, phenotypic, and genetic changes. These properties are also modulated by the medium formula employed to expand the cell populations. While aged MSCs maintain a certain ability to differentiate toward the adipogenic lineage regardless of the expansion condition, their osteogenic potential is significantly impaired by senescence. For osteogenic induction, one must first consider MSCs at early passages, and use of the αMEM-based proliferation medium may yield longer osteogenesis and better quality. The extent of the observed changes attributed to in-vitro senescence, however, may be affected by several factors such as cell origin, donor age, and patient pathologic conditions which can yield MSCs with varied properties and functionalities and thus needs to be further evaluated. Collectively, this study provides insightful information for future basic science research and clinical applications leading to development of innovative MSC therapies for various diseases.

## Additional file

Additional file 1: **Figure S1.** Relative telomere length of MSCs passaged in the DMEM-based (C) or αMEM-based (D) expansion medium. Relative telomere length was measured by SYBR Green qPCR amplification of telomere repeats and a single copy gene 36B4 [39]. Briefly, MSCs at different passage numbers were lysed overnight in a solution composed of 1% sodium dodecyl sulfate, 10 mM Tris-hydrochloride, 1 mM ethylenediaminetetraacetic acid, 100 mM sodium chloride, and 300 mg/mL proteinase K, followed by 2-h digestion with HinfI and RsaI at 37 °C to extract genomic DNA. Isolated DNA (35 ng/sample) was then mixed with SYBR Green PCR Master Mix and telomere or 36B4 primers. The final concentrations of the primers were as follows: telomere 1, 5’-GGTTTTTGAGGGTGAGGGTGAGGGTGAGGGTGAGGGT-3’, 270 nM; telomere 2, 5’-TCCCGACTATCCCTATCCCTATCCCTATCCCTATCCCTA-3’, 900 nM; 36B4u, 5’-CAGCAAGTGGGAAGGTGTAATCC-3’, 300 nM; 36B4d, 5’-CCCATTCTATCATCAACGGGTACAA-3’, 500 nM. The telomere and 36B4 PCRs were carried out in separate plates and the reactions consisted of an initial enzyme activation for 10 min at 95 °C, followed by 40 cycles of 15 s at 95 °C and 2 min at 54 °C for telomere PCR or 40 cycles of 15 s at 95 °C and 1 min at 58 °C for 36B4 PCR. Standard curves (A, B) generated from serial dilution of DNA (12.5 to 100 ng) extracted from the telomerase-positive K562 cell line were also included in PCRs and used to determine the quantities of telomere repeats (T) and 36B4 (S) from the corresponding Ct values of each sample. Relative telomere length was estimated as the T-to-S ratio. A decreasing trend in telomere length was observed over time in MSCs cultivated with either expansion medium. Specifically, the T-to-S ratio reduced from 1.53 at P3 to 0.49 at P8 in the DMEM group (C) and from 1.72 at P3 to 0.70 at P8 in the αMEM group (D). Numbers represent the mean values. *n* = 4; *p < 0.05, versus the corresponding P8-MSCs passaged under the same expansion condition. (PDF 711 kb)

## Abbreviations

αMEM: Minimum essential medium alpha
ALP: Alkaline phosphatase
Col I: Type I collagen
CPDT: Cell population doubling time
Ct: Cycle threshold
DMEM: Dulbecco’s modified Eagle’s medium
ECM: Extracellular matrix
FBS: Fetal bovine serum
FGF-2: Basic fibroblast growth factor
GAPDH: Glyceraldehyde-3-phosphate dehydrogenase
HSC: Hematopoietic stem cell
LPL: Lipoprotein lipase
MSC: Mesenchymal stem cell
NCPD: Number of cell population doubling
P: Passage
PBS: Phosphate-buffered saline
PPARγ: Peroxisome proliferator-activated receptor γ
qPCR: Real-time polymerase chain reaction
RUNX2: Runt-related transcription factor 2
