# Supplementary material for: Changes in phenotype and differentiation potential of human mesenchymal stem cells aging in vitro
Source: Stem Cell Res Ther. 2018 May 11;9:131. doi: 10.1186/s13287-018-0876-3 (PMC5948736; doi:10.1186/s13287-018-0876-3)
Supplement: Supplementary file 1 — Figure S1. Relative telomere length of MSCs passaged in the DMEM-based (C) or αMEM-based (D) expansion medium. Relative telomere length was measured by SYBR Green qPCR amplification of telomere repeats and a single copy gene 36B4 [39]. Briefly, MSCs at different passage numbers were lysed overnight in a solution composed of 1% sodium dodecyl sulfate, 10 mM Tris-hydrochloride, 1 mM ethylenediaminetetraacetic acid, 100 mM sodium chloride, and 300 mg/mL proteinase K, followed by 2-h digestion with HinfI and RsaI at 37 °C to extract genomic DNA. Isolated DNA (35 ng/sample) was then mixed with SYBR Green PCR Master Mix and telomere or 36B4 primers. The final concentrations of the primers were as follows: telomere 1, 5’-GGTTTTTGAGGGTGAGGGTGAGGGTGAGGGTGAGGGT-3’, 270 nM; telomere 2, 5’-TCCCGACTATCCCTATCCCTATCCCTATCCCTATCCCTA-3’, 900 nM; 36B4u, 5’-CAGCAAGTGGGAAGGTGTAATCC-3’, 300 nM; 36B4d, 5’-CCCATTCTATCATCAACGGGTACAA-3’, 500 nM. The telomere and 36B4 PCRs were carried out in separate plates and the reactions consisted of an initial enzyme activation for 10 min at 95 °C, followed by 40 cycles of 15 s at 95 °C and 2 min at 54 °C for telomere PCR or 40 cycles of 15 s at 95 °C and 1 min at 58 °C for 36B4 PCR. Standard curves (A, B) generated from serial dilution of DNA (12.5 to 100 ng) extracted from the telomerase-positive K562 cell line were also included in PCRs and used to determine the quantities of telomere repeats (T) and 36B4 (S) from the corresponding Ct values of each sample. Relative telomere length was estimated as the T-to-S ratio. A decreasing trend in telomere length was observed over time in MSCs cultivated with either expansion medium. Specifically, the T-to-S ratio reduced from 1.53 at P3 to 0.49 at P8 in the DMEM group (C) and from 1.72 at P3 to 0.70 at P8 in the αMEM group (D). Numbers represent the mean values. n = 4; *p < 0.05, versus the corresponding P8-MSCs passaged under the same expansion condition. (PDF 711 kb) [file 13287_2018_876_MOESM1_ESM.pdf]

Changes in Phenotype and Differentiation Potential of Human Mesenchymal Stem Cells Aging In Vitro

Yueh-Hsun Kevin Yang, Courtney R. Ogando, Carmine Wang See, Tsui-Yun Chang, Gilda A. Barabino

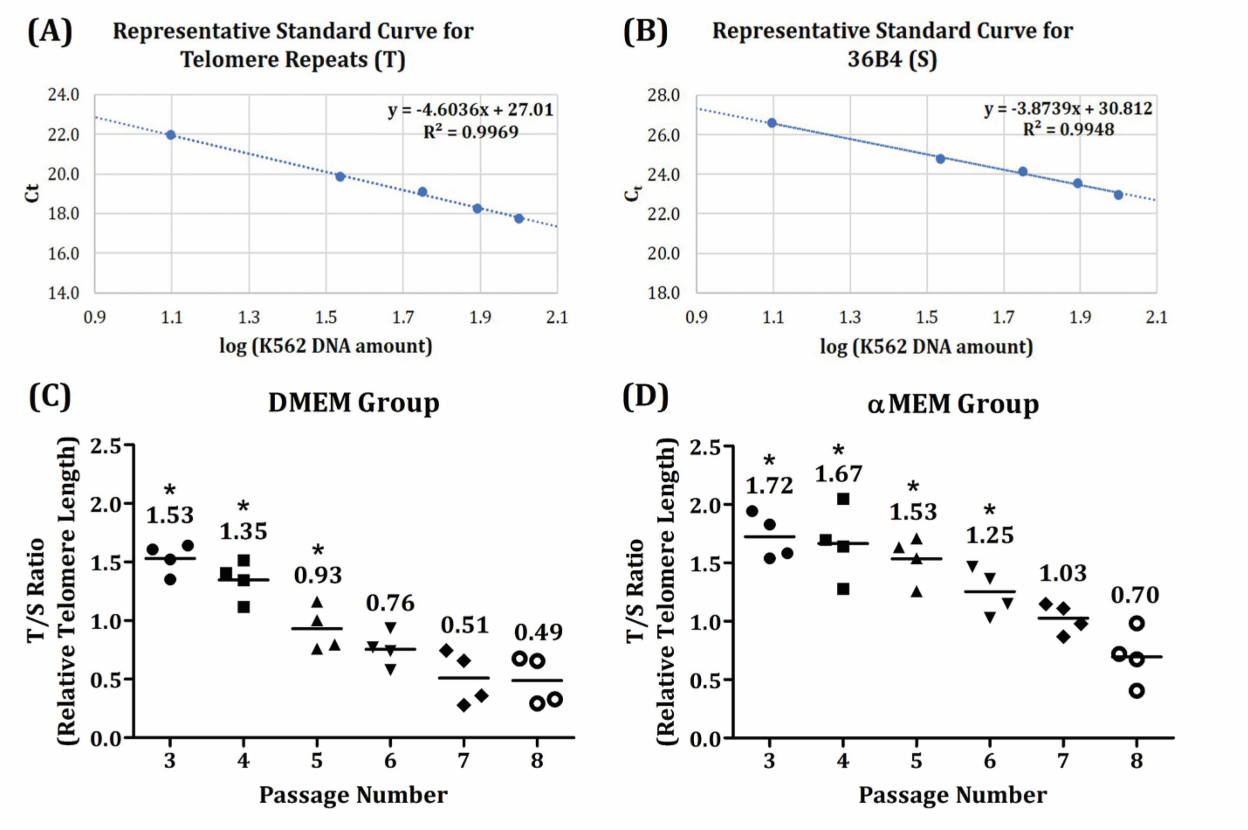

Additional File 1: Figure S1. Relative telomere length of MSCs passaged in the DMEM-based (C) or αMEM-based (D) expansion medium. Relative telomere length was measured by SYBR Green qPCR amplification of telomere repeats and a single copy gene 36B4 [39]. Briefly, MSCs at different passage numbers were lysed overnight in a solution composed of 1% sodium dodecyl sulfate, 10 mM Tris-hydrochloride, 1 mM ethylenediaminetetraacetic acid, 100 mM sodium chloride and 300 mg/mL proteinase K, followed by 2-hour digestion with HinfI and RsaI

at 37°C to extract genomic DNA. Isolated DNA [35 ng/sample] was then mixed with SYBR Green PCR Master Mix and telomere or 36B4 primers. The final concentrations of the primers were as follows: telomere 1 [5'-GGTTTTTGAGGGTGAGGGTGAGGGTGAGGGTGAGGGT-3']: 270 nM; telomere 2 [5'-TCCCGACTATCCCTATCCCTATCCCTATCCCTATCCCTA-3']: 900 nM; 36B4u [5'-CAGCAAGTGGGAAGGTGTAATCC-3']: 300 nM; 36B4d [5'-CCCATTCTATCATCAACGGGTACAA-3']: 500 nM. The telomere and 36B4 PCRs were carried out in separate plates and the reactions consisted of an initial enzyme activation for 10 minutes at 95°C, followed by 40 cycles of 15 seconds at 95°C and 2 minutes at 54°C for telomere PCR or 40 cycles of 15 seconds at 95°C and 1 minute at 58°C for 36B4 PCR. Standard curves (A, B) generated from serial dilution of DNA [12.5 to 100 ng] extracted from the telomerase-positive K562 cell line were also included in PCRs and used to determine the quantities of telomere repeats [T] and 36B4 [S] from the corresponding C_t values of each sample. Relative telomere length was estimated as T-to-S ratio. A decreasing trend in telomere length was observed over time in MSCs cultivated with either expansion medium. Specifically, T-to-S ratio reduced from 1.53 at P3 to 0.49 at P8 in the DMEM group (C) and from 1.72 at P3 to 0.70 at P8 in the αMEM group (D). Numbers represent the mean values. *indicates statistical significance in comparison with the corresponding P8-MSCs passaged under the same expansion condition. n = 4; $p < 0.05$.
